# Characteristics of domestic violence perpetrators with dementia from police records using text mining

**DOI:** 10.3389/fpsyt.2024.1331915

**Published:** 2024-05-14

**Authors:** Sharon Reutens, George Karystianis, Adrienne Withall, Tony Butler

**Affiliations:** ^1^ School of Population Health, Faculty of Medicine, University of New South Wales, Sydney, NSW, Australia; ^2^ School of Psychology, Faculty of Science, University of New South Wales, Sydney, NSW, Australia; ^3^ Ageing Futures Institute, University of New South Wales, Sydney, NSW, Australia

**Keywords:** dementia, text mining, domestic violence, older offenders, offenders, mental health, young-onset dementia

## Abstract

**Aim:**

Few studies have examined the characteristics of domestic violence (DV) committed by people with dementia. We provide an overview of DV perpetrated by people with dementia in the community based on police reports of attendances at DV events.

**Method:**

A text mining method was used on 416,441 New South Wales (NSW) police narratives of DV events from January 2005 to December 2016 to extract information for Persons of Interest (POIs) with mentions of dementia.

**Results:**

Events involving those with dementia accounted for a relatively low proportion of total DV events (<1%). Of the 260 DV events with a dementia mention for the POI, the most common abuse types were assault (49.7%) and verbal abuse (31.6%). Spouses were the largest group of victims (50.8%) followed by children (8.8%). Physical abuse was common, occurring in 82.4% of events, but injuries were relatively mild. Although weapons were infrequently used, they were involved in 5% of events, mostly by POIs aged 75 years and older. Similarly, the POIs were mainly aged 75+ years (60%), however the proportion of those aged <65 was relatively high (20.8%) compared to the reported prevalence of dementia in that age group.

**Conclusions:**

This study demonstrates that some cases of DV perpetrated by people with reported dementia are significant enough to warrant police involvement. This highlights the need to proactively discuss the potential for violence as part of the holistic management and support family members, particularly those caring for people with young-onset dementias.

## Introduction

The term “dementia” encompasses neurodegenerative and non-degenerative conditions characterized by cognitive and functional decline. The global prevalence of dementia in people aged over 50 years has been calculated to be 697 per 10,000 and it is more common in females than males (1). Projected increases in the global prevalence of dementia are attributed to both population growth and ageing, and are estimated to rise from 57.4 million cases in 2019 to 152.8 cases in 2050 (2).

This rise in dementia cases is likely to result in a concomitant increased prevalence of dementia-related neuropsychiatric symptoms, such as aggression, irritability, depression, and psychosis. These symptoms can result from the neuroanatomical circuits affected by dementia pathology and/or may be a way for the person with dementia to express their unmet medical, social or environmental needs (3). In particular, aggression has been widely reported in cross-sectional studies of people with dementia (4–6), with one study reporting that 30% of 682 community-dwelling people diagnosed with dementia or mild cognitive impairment demonstrated agitation or aggression on the Neuropsychiatric Inventory (7). Further, a study that followed 99 people diagnosed with Alzheimer’s disease who exhibited signs of aggressive behavior over a 10-year period revealed that physical aggression occurred in 61% of cases (8).

Such neuropsychiatric symptoms potentially increase the risk of a person with dementia committing acts of domestic violence (DV) as defined in the New South Wales (NSW, Australia) Crimes (Domestic and Personal Violence) Act 2007 No 80 as any “*physical, sexual, financial, emotional or psychological abuse perpetrated within a current or past intimate partner and family relationship”*. The Act further states that this can apply to residents in the same residential facility, paid carers, extended family including step-family members, dependents, and for Aboriginal peoples, their kin. As such, the term “domestic” refers to the relationship between the perpetrator and victim rather than the location of the offence itself and may be used when the perpetrator is living or has lived in the same household as the other person.

Intimate partner violence is defined as violence between current or previous cohabiting partner, current or ex-boyfriend/girlfriend or date (9), affecting more than one quarter (27%) of women aged between 15 to 49 years (10) and has a lifetime prevalence of 16.5%- 54.5% in women aged 45 years and over (11). Male victimization has been found to occur as frequently as violence against women in some studies, but men are less likely to seek assistance and less likely to incur serious injuries (12). Research to date indicates that although physical abuse of intimate partners might decline with age, emotional abuse does not (13–15). However, profiles of intimate partner violence offenders are typically based on self-report surveys of current or former intimate partner victims (16).

A complicating factor is the difference in terminology that can be applied to violence committed by older people with dementia, particularly if the victim is similarly older and frail and/or cognitively impaired. In practice, the violence can be construed as DV, its subset, elder abuse (defined as “acts of omission or commission that result in psychological, physical, financial or sexual harm to a person” perpetrated by a person in a position of trust) (17) or as neuropsychiatric symptoms of dementia (“a heterogenous group of non-cognitive symptoms that are almost ubiquitous in dementia”) (17, 18) with important implications for interventions for the perpetrator and the victim. If the violence is viewed as a symptom of dementia, the perpetrator can be managed by the medical system. However, criminal charges can ensue if it is classified as DV. The distinction between these behaviors has not been defined in law or in a clinically setting, thus the decision to proceed with charges is made by the arresting police officer.

The police are often the first to respond to DV events in the community. While attending, they write detailed reports that describe the event recording salient details of the incident such as perpetrator and victim characteristics (e.g., sex, age), premises type and observable injuries. These reports have been shown to be a valuable source of information on DV (19, 20), providing direction for further clinical research due to their provision of an independent portrait of events that involve aggression within the community - irrespective of the underlying contributions. These text records enable identification of the characteristics of domestic violence perpetrators (hereafter referred to as Persons of Interest (POIs) i.e., individuals involved in an event that have been accused or charged for perpetrating DV related crimes) that can inform further research, clinical screening, and interventions. Police records enable characteristics of DV events to be described (19) based on the setting (e.g., nursing homes) (21), specific mental health conditions (e.g., autism) (22) and abuse types (e.g., non-fatal strangulation, coercive control) (23, 24), and population sub-groups (25).

This exploratory study aims to profile DV POIs with a reported dementia mention in the police records to determine their characteristics and any associated factors to provide a basis for future research or modes of intervention.

## Methods

### Data

The police records comprise structured data (called fixed fields) and text narratives. The fixed fields contain demographic and other information related to the individuals involved in the domestic dispute such as the sex, postcode, ethnicity, premises type, and any charged offence(s). The text narratives are in the form of unstructured text and detail the circumstances of the event, including the cause of the event, persons present, any substance and/or alcohol abuse, the mental health status of the POI and victim, victim injuries, type of abuse, and any actions taken by the police.

The police records were flagged on their database with one of the three values in the fixed fields: “domestic” as the type of the offence; “domestic violence related”, or the relationship status between the victim and the perpetrator was described as any of the following: “spouse/partner (including ex-spouse/ex-partner)”, ‘boy/girlfriend (including ex-boy/ex-girlfriend)”, “parent/guardian (including step/foster)”, “child (including step/foster)”, “sibling”, “other member of family (including kin)”, or “carer (including paid carers)”. The records covered the following offence categories: assault, breach of Apprehended Domestic Violence Orders (ADVO – a restraining or protection order that places prohibitions and/or restrictions on a persons accused of DV), homicide, malicious damage to property, and offences against a person such as kidnapping, abduction or harassment.

### Cohort definition

For this research we used a data subset from a text mining study on 492,393 DV events from the NSW Police Force’s Computerized Operational Policing System (COPS) database covering the period of January 2005 to December 2016. Extracted results from 64,587 events involving multiple perpetrators or victims were excluded from the analysis as it was not possible to deduce to whom the extracted mental health mention referred to (i.e., if an abuse type was extracted in an event involving two POIs, it is not certain if it was conducted by the first or the second POI), leaving 416,44 events for our analysis.

Our cohort definition included DV events with POIs aged 18 years or over at the time of the report who were living in the community whose narratives had recorded a mention by the police of an ICD-10 diagnosis of dementia or a dementia sub-type (i.e., G31.0 frontotemporal dementia, G10 Huntington’s disease, F01 vascular dementia, F00.9 Alzheimer’s dementia) (27). This resulted in 260 events involving POIs over the age of 18 years with one of the aforementioned diagnoses. Its methodology to identify key information from police narratives such as abuse types, victim injuries and mental health mentions for POIs and victims is summarized below (19).

### Text mining approach

We used General Architecture for Text Engineering (GATE) (26), a family of open-source text analysis tools and processes that supports the development and application of automated approaches to identify information on mental illnesses in POIs and victims, abuse types conducted by POIs, and victim sustained injuries (26). Text mining permits the examination of large amounts of material, which, if manually examined, would be onerous and time-consuming. Our approach was based on rules that rely on common syntactical patterns observed in text which suggest the presence of a mental illness mention (e.g., “the POI has a form of dementia”), abuse types (“perpetrator has kicked the victim in the chest”) and victim injuries (“victim sustained several lacerations on her arm”). A sample of 200 DV narratives were used to design the rules.

Dictionaries that contained terms, common abbreviations and synonyms for mental illnesses, abuse types and injuries were manually engineered and combined with our rules. The syntactical patterns use: (1) frozen lexical expressions as anchors for certain elements that are built through specific verbs, noun phrases, and prepositions (e.g., “person of interest is suffering from”); and (2) semantic place holders (identifiable through the application of the manually crafted dictionaries) that indicate the presence of a mental illness, abuse type or an injury.

In this example (“perpetrator has frontotemporal dementia”), “frontotemporal dementia” is a mental illness mention that we seek to recognize. “Perpetrator has” is a frozen syntactical expression indicating a mental illness mention and “frontotemporal dementia” gets a match in the dictionary of mental illnesses terms.

The method was evaluated in a random sample of 100 DV narratives for the mental illness mentions with an average 92% precision (i.e., the percentage of correctly identified mental illness mentions against the total number of identified mental illness mentions, a denominator that includes both true positives and false positives) for both mental illnesses mentions for perpetrators (97.5% precision) and victims (87.1% precision), and 90.2% for abuse types and 85.0% precision for victim injuries. A description and evaluation of the methodology applied to this dataset has been published in detail (19, 20).

We classified the extracted mental health conditions according to the International Classification of Diseases and Related Health Problems (ICD-10) (27). Abuse types were categorized into nine categories under 46 different types and 17 types of injuries were recorded.

### Statistical analysis

Descriptive statistics were generated using IBM SPSS Statistical package version 27 (28). Demographics were obtained for POIs aged between 18 and 54 years, 55 to 64 years, 65 to 74 years and 75 years and older.

### Ethics

Permission to access data was given by the NSW Police Force following ethics approval being granted by the University of New South Wales Human Research Ethics Committee (reference HC16558).

## Results

### Demographics

Overall, 260 events had a mention of dementia associated with a POI (**Table 1**). POIs with a dementia mention in the police records were predominantly male (195; 75.0%) (**Table 1**). These proportions were reversed with the respective victims, with the majority being female (74.2%; 193). Most of the POIs were aged 75 years and over (156; 60.0%), however 20.8% (n=54) of events involved POIs aged under 65 years, that is those who had young onset dementia (YOD). In contrast, about two thirds of victims were aged under 75 years (67.3%) and about one quarter (n=40; 25.6%) were aged under 55 years (**Table 2**).

**Table 1:** Sex and age bands of victims and POIs.

Sex	Persons of Interest		Victims	
	Number of events	%	Number of events	%
Female	65	25	193	74
Male	195	75	63	24
Unknown	0	0	4	2
**Total**	**260**	**100**	**260**	**100**
Age group (years)				
8-54	26	10	40	26
55-64	28	11	31	20
65-74	50	19	34	22
≥75	156	60	51	33
**Total**	**260**	**100**	**156**	**101**

Bold values are the total number of events.

**Table 2:** Relationship between the POI and the victim in 225 events (more than one relationship type might be recorded in an event).

POI’s relationship with victim	Number of events (n=225)	%
Spouse/partner	132	59
Parent/guardian of the victim	23	10
Other family member	22	10
Other known person	17	8
Carer	15	7
Household member	10	4
Child of victim	9	4
Sibling	3	1
Ex-spouse	3	1
Not known to victim	2	1
Boyfriend/girlfriend including ex	2	1

Over half of the recorded events reported a current “spouse/partner” as the relationship between the victim and POI (n=132; 58.7%), with other victims mainly comprising other family members of the person with dementia (25.3%). This included children, other family members, and parents. Non-family members, including carers but excluding former intimate partners, were cited as victims in 19.6% of cases (**Table 3**).

**Table 3:** Abuse types in 260 DV police narratives involving a POI dementia mention from January 2005 to December 2016 (more than one abuse type might be recorded in an event).

Abuse type	Number of events (n=193)	%
Assault^$^	96	50
Property damage	61	32
Verbal abuse	60	31
Intimidation	58	30
Punching^%^	46	24
Grabbing^%^	34	18
Pushing^%^	32	17
Kicking^%^	15	8
Scratching^%^	11	6
Physical restraint^%^	9	5
Choking^%^	6	3
Slapping^%^	6	3
Hitting with a weapon^%^	6	3
Breach of restraining order	5	3
Miscellaneous	5	3
Pulling^%^	5	3
Spitting^%^	5	3
Stabbing^%^	5	3
Throwing an object to harm the victim^%^	5	3
Physically blocking the victim’s space^%^	4	2
Hair pulling ^%^	4	2
Lunging towards the victim	4	2
Self-harm	4	2
Withholding of personal effects	2	1
Shaking^%^	2	1
Social isolation	1	1
Biting^%^	1	1
Chasing the victim	1	1
Putting victim in headlock^%^	1	1
Stalking	1	1
Throwing the victim^%^	1	1

$Includes unspecified physical abuse. %Specific physical abuse type.

### Mental health conditions

Most DV events included mentions of unspecified dementia (n=216; 83.1%). Thirty-two events referred to Alzheimer’s disease (12.3%), six events (2.3%) to Huntington’s dementia, five (1.9%) to frontotemporal dementia and one (0.4%) to vascular dementia.

For some POIs, there was mention of co-existing mental health conditions. The most common co-existing mental health illness extracted was major depressive disorder (n=10; 3.8%), psychotic conditions were mentioned for 2.7% (n= 7; i.e., schizophrenia, delusional disorder, brief psychotic disorder), a further 2.7% for alcohol abuse (n=7), and persistent mood disorder (n=6; 2.3%).

### Abuse types and injuries

Abuse types were recorded in three-quarters (n=193; 74.2%) of DV events involving dementia detailed in **Table 4**. Both physical and non-physical (e.g., verbal, emotional) abuse was common, especially physical abuse, which occurred in 82.4% (n=159) of the abuse events. Non-physical and physical abuse co-occurred in 108 (56%) events. Threats were relatively uncommon, (n=14; 7.3%), with most of these (13; 92.9%) related to threats to kill by the POI. Most of the recorded abuse types were unspecified physical abuse termed as assaults (n=96; 49.7%) and property damage (61, 31.6%) (**Table 4**), followed by verbal abuse (60; 31.1%) and intimidation (58; 30.1%). Lesser-known forms of abuse, such as social isolation, victim chasing and stalking, had the lowest event prevalence (n=1; 0.5%).

**Table 4:** Victim Injuries in 260 DV police narratives involving a POI dementia mention from January 2005 to December 2016 (more than one injury type might be recorded in an event).

Injury type	Number of events (n=85)	%
Red area	17	20
Cut(s)	16	19
Bruise(s)	15	18
Soreness	12	14
Swelling	10	12
Miscellaneous	6	7
Lump	3	4
Fracture(s)	3	4
Bleeding	2	2
Stab wound(s)	1	1
Grazing	1	1
Periorbital hematoma	1	1

Despite the frequent use of violence, physical injuries were only documented in one third (n= 85; 32.6%) of the 260 events (**Table 4**). Whilst most of the recorded injuries were in the milder spectrum (e.g., cuts, swelling, redness of skin), one of the events resulted in a charge of homicide.

Weapons were infrequently involved, with 13 events out of 260 (5%) involving 22 different weapons. Sharp objects and knives were the most-used weapons (n=18; 81.8%). Most events (9/13, 69.2%) involving weapons occurred in POIs aged 75 years and over.

### Location

Most DV events occurred in private residences (192; 73.9%), with only 6 of the 260 events (2.3%) occurring in outside locations (e.g., footpath, street). Forty-six events (17.7%) occurred in an aged care facility, one event (0.4%) occurred in a hospital, while the remaining were recorded in lodges (1; 0.4%), hostels (3; 1.2%) and unspecified locations (11; 4.2%).

### Charges

A total of 282 charges were made by the police, noting that one event can result in multiple charges. The high percentage of violent acts in this group was reflected in the large proportion of charges made in relation to assault (n=215; 82.7%), 16 charges regarding property damage (6.2%), and one charge of homicide (0.4% of events).

## Discussion

These data provide an overview of DV cases in which attending police officers recorded instances of dementia linked to a POI. Our findings highlight key insights of situations and parties at risk of DV in the context of dementia through the ages of the POIs, their gender, victims, and types of violent offending.

We found that POIs were predominantly male (75%) and mostly aged 75 years and older (60%). However, about one in five events involved POIs with dementia who were aged under 65, which constitutes young onset dementia (YOD). This is greater than the global prevalence of YOD, which comprises 8.7% of all dementia cases and has been calculated at a rate of 119 per 100,000 of population (29). These findings support the literature regarding an increased burden of caring for people with YOD, who are relatively physically healthy when compared to people with late onset dementias (30, 31). Within young onset dementia, there is a higher rate of dementias associated with increased behavioral and psychological symptoms of dementia, such as aggression, loss of empathy, and disinhibition (30, 32). This includes Huntington’s disease and the frontotemporal dementias, which are conditions characterized by prominent executive dysfunction and behavioral features that may lead to an increased risk of violent offending (33, 34). Increased caregiver burden has been associated with executive dysfunction in YOD (30), perhaps because the features of impulsivity and lack of empathy may increase the possibility of reactive violence (35) and inappropriate language towards the carer. People with YOD tend to remain in the home for longer than those with late onset dementias, and, unlike those with late onset dementia, aggression does not predict institutionalization for this group (36). Our findings suggest that family members of people with YOD who live at home sometimes require police assistance to manage physically capable people with behaviors that may escalate to violence. These family members are a group that should receive increased education regarding behavioral changes associated with dementia and how these may reflect unmet needs, as well as methods to safely de-escalate conflict.

Whilst unspecified dementia was the most cited category, which is to be expected given the information was derived from police narratives, several subtypes of dementia were mentioned. These included Alzheimer’s disease, Huntington’s disease and frontotemporal dementia. Co-existing mental health conditions, predominantly major depressive disorder (3.8%), were also reported, although the prevalence of these conditions was relatively low compared to published studies of psychiatric comorbidity in dementia (7, 37, 38). For instance, in the Cardiovascular Health Study conducted in the community, clinical levels of depression and anxiety were found in 16.0% and 9.7% of the 362 participants with dementia respectively, with a further 10.5% having clinically significant delusions and 5.0% with clinically significant hallucinations (7).

Intimate partners and other family members were shown to be the most common victims, accounting for 92.2% of events. This indicates that family members, and spouses in particular, are an at-risk group and require support to manage problem behaviors. Caregivers may be reluctant to report DV as they fear it reflects negatively on the person with dementia, but family caregiver training has been shown to reduce caregiver burden and challenging behaviors (39, 40). Service delivery for the management of behaviors of concern in dementia in Australia currently varies due to limited resources, particularly in rural and remote areas (18).

Overall, the main driver for requesting police assistance was violence to people and property, resulting in most of the charges pertaining to assault (82.7%). Physical injuries were generally minor, but the single charge of homicide is a reminder that although seemingly frail, people with dementia can still act with fatal force, particularly if directed at an equally frail victim (41).

Kang & Lynch have alluded to the nuances involved in DV victims’ decisions to call the police, with age and type of familial relationship to the perpetrator (e.g., spouse, child of POI) factoring into decision-making (42). They found that, in general, older victims (aged 55 and over) were less likely to call for assistance if the POI was a spouse compared to younger victims, possibly because of greater dependency on the spouse (42). Similarly, victims of people with dementia in a domestic setting may be less inclined to involve police due to a belief that the POIs behavior is influenced by their dementia or because they have already endured years of abuse (17, 43). We were unable to identify any studies into the impetus (or failure) to involve police in cases where the perpetrator suffered from dementia, thus further research is required in this area. Nonetheless, interpretation of the event data should take into account these underlying biases and the possibility of underreporting by victims of older POIs with dementia. Our findings are consistent with most DV studies in this age group showing victims are mainly female (16, 44). Notably, one in four victims in this study were male, possibly family members or carers, suggesting that the internal and external pressures against disclosure and challenges to masculinity, which have been cited as barriers for seeking help in males facing intimate partner violence (45), might not be as relevant in cases involving POIs with dementia.

The relatively low proportion of events situated at aged care facilities is interesting given research that has indicated a high prevalence of aggression in such facilities (46, 47). For instance, a longitudinal study utilizing four-monthly assessments of 56 people in a nursing home until death found that 89% of residents demonstrated verbal aggression and 61% physical aggression over the course of the follow-up period (8). Thus, it is likely that aged care staff have more strategies to manage aggression and more likely to tolerate aggression before calling for outside assistance. This finding highlights that further research into the availability of support mechanisms to family members of people with dementia in home care is a priority. Timely intervention by multidisciplinary health services providing social support, respite, medical assessment and management of problem behaviors could reduce the rates of violence and assist people with dementia to stay in their homes for longer.

These cases are all derived from events where police were called to the scene. They were deemed severe enough by participants or witnesses to warrant police intervention but also show that police are front line workers interacting with people with dementia. While it is not within the remit of police officers to be able to diagnose dementia, they would benefit from a greater understanding of the types of behaviors and triggers for people with dementia-associated aggression and irritability, which could assist in de-escalation of an acute situation. Research and cooperation between clinicians and police are required to develop strategies to manage and disarm a dangerous, physically frail person without causing serious injury or death.

This field is hampered by a lack of a cohesive conceptual framework for violence in dementia; that is, not all violence is offending but the perception often depends on whether the behavior is regarded as arising from the dementia or manifesting from characterological traits (13, 17). Research in this area has also predominantly focused on victims of intimate partner violence or elder abuse (48), with knowledge about perpetrators of violence mostly gleaned from the victim’s report of the type of abuses and characteristics of the perpetrator (16, 49, 50). This study provides an overview of aggression perpetrated by older adults with dementia, irrespective of the lens through which it is viewed. Aggression and violence perpetuated by people with dementia are usually construed as a symptom of the dementia, with little consideration of personality or aggression predating the onset of dementia (46). This presumption may place family members of people with dementia at risk of DV if not discussed with a clinician. Our findings have also shown that it would be beneficial to extend DV screening to other family members in addition to spouses.

## Limitations

Caution is required when interpreting and generalizing these findings. In particular, the mentions of the various dementia forms and co-existing mental illnesses have not been clinically validated. Attending police officers who respond to a DV event prioritize the victim’s safety and note down observable information such as sustained injuries, property damage or alcohol and other visible substance abuse. They are not trained to inquire about the nuanced nature of dementia and its subtypes. Police officers rely on the reports of victims, POIs and witnesses, such that diagnoses (and other important information) might not be included if they are not mentioned. Thus, our subset of 260 events involving cases of dementia are likely to be an underestimate. Additionally, we were unable to ascertain the age of onset of cognitive impairment using this. Consequently we could not rule out the possibility that some offenders in the age group 65-74 did not have YOD, and our findings may underrepresent those with YOD. Further, despite the accuracy of the text mining method in the extraction of key information from DV event narratives (19), some of the mental illness, abuse type and victim injuries might have been missed, mainly due to the complexities of the English language and the scope of the utilized dictionaries (19, 26).

However, this study provides important information about aggression in the context of DV events perpetrated by people with dementia. Unlike studies that depend on active recruitment of patients with dementia in clinical settings or in the community, this data is not hampered by recruitment biases.

## Conclusions

We demonstrated that examining DV police records can provide valuable information regarding a better understanding of POIs who have a reported dementia mention. Despite the apparent frailty of POIs with dementia, DV events involving such individuals are serious, potentially fatal, and mainly affect spouses (51), who may be particularly vulnerable by virtue of their advanced age. Spouses and broader family members would benefit from knowledge regarding available support and de-escalation strategies if experiencing DV. Although existing services (e.g., Dementia Support Australia) offer scaled assistance for people with dementia, it is recommended that police officers undertake specific collaborations and cross-disciplinary police training to coordinate the management of physically strong people with dementia who may show aggressive behaviors in residential settings and aged care facilities. This initiative could involve dementia education, joint callouts and facilitation of direct referral pathways to specialist dementia behavior management teams. For effective delivery, an agreed-upon definition differentiating domestic violence from elder abuse and research into the underlying factors driving dementia-related behaviors is required. Additionally, further research into the experiences of victims of domestic abuse conducted by people with dementia would be useful for the development of support mechanisms, early prevention and intervention strategies, and valid screening tools.

## Data availability statement

The original contributions presented in the study are included in the article/supplementary material, further inquiries can be directed to the corresponding author.

## Ethics statement

The studies involving humans were approved by the University of New South Wales Human Research Ethics Committee (reference HC16558). The studies were conducted in accordance with the local legislation and institutional requirements. Written informed consent for participation was not required from the participants or the participants’ legal guardians/next of kin in accordance with the national legislation and institutional requirements.

## Author contributions

SR: Conceptualization, Formal analysis, Methodology, Writing – original draft, Writing – review & editing. GK: Data curation, Formal analysis, Methodology, Software, Supervision, Writing – review & editing. AW: Conceptualization, Methodology, Supervision, Writing – review & editing. TB: Conceptualization, Methodology, Supervision, Writing – review & editing.
